# Hypnosis in the operating room: are anesthesiology teams interested and well-informed?

**DOI:** 10.1186/s12871-023-02229-3

**Published:** 2023-08-24

**Authors:** Sonia Zaccarini, Aurore Fernandez, Adriana Wolff, Lennart Magnusson, Benno Rehberg-Klug, Sina Grape, Patrick Schoettker, Chantal Berna

**Affiliations:** 1 Center for Complementary and Integrative Medicine, Department of Anesthesiology, Lausanne University Hospital (CHUV), and Lausanne University, Rue du Bugnon 46, Lausanne, 1011 Switzerland; 2 Pain Center, Department of Anesthesiology, Lausanne University Hospital (CHUV), Lausanne, Switzerland; 3 Department of Anesthesiology, University of Geneva Hospitals, Geneva, Switzerland; 4 Department of Anesthesiology, Cantons Hospital of Fribourg, Fribourg, Switzerland; 5 Department of Anesthesiology, Valais Hospital, Sion, Switzerland

**Keywords:** Hypnosis, Hypnoanalagesia, Anesthesiology, Implementation, Operating room

## Abstract

**Background:**

Hypnosis can be a beneficial complementary anesthesia technique for a variety of surgical procedures. Despite favorable scientific evidence, hypnosis is still rarely used in the operating room. Obstacles to implementation could be a lack of interest or training, misconceptions, as well as limited knowledge amongst anesthesiology teams. Hence, this study aimed to assess the interest, training, beliefs, and knowledge about hypnosis in the operating room staff.

**Design:**

A questionnaire with 21-items, based on a prior survey, was set up on an online platform. The medical and nursing anesthesiology staff of four Swiss academic and large regional hospitals (N = 754) were invited to participate anonymously through e-mails sent by their hierarchy. Results were analyzed quantitatively.

**Results:**

Between June, 2020 and August, 2021 353 answers were collected (47% response rate). Most (92%) were aware that hypnosis needs specific training, with 14% trained. A large majority of the untrained staff wished to enroll for conversational hypnosis training. There was a strong agreement for hypnosis playing a role in anesthesia. Nevertheless, many of these professionals believed that hypnosis has a limited field of action (53%) or that it would be too time consuming (33%). The reduction of misconceptions was based more on exposure to hypnosis than on training.

**Conclusion:**

Overall, anesthesia providers’ attitude was in favor of using hypnosis in the operating room. Misconceptions such as a prolongation of the procedure, alteration of consent, lack of acceptability for patients, and limited indications were identified as potential barriers. These deserve to be challenged through proper dissemination of the recent scientific literature and exposure to practice.

**Supplementary Information:**

The online version contains supplementary material available at 10.1186/s12871-023-02229-3.

## Introduction

Medical hypnosis is a relational care technique that has shown its usefulness in the operating room (OR) [1]. Formal hypnosis includes codified stages starting with an induction [2]. Conversational hypnosis [3] uses verbal suggestions and other hypnotic communication tools without a formal hypnotic induction. Both of these related techniques - referred to as “hypnoanalgesia”- can be used perioperatively as part of a multimodal management plan [4, 5]. The effectiveness of hypnoanalgesia is well demonstrated during different surgical or interventional procedures [6–9] Meta-analyses highlight hypnoanalgesia as a safe and effective complementary technique, with benefits on pain and stress compared to standard treatment or attention control [10], as well as significant positive effects on medication consumption, recovery, and surgical procedure duration [11].

Nevertheless, the use of hypnoanalgesia remains rare. Promoting the implementation of a new medical practice requires an understanding of health providers’ knowledge regarding the supporting evidence, followed by efficient education to eliminate misconceptions [12–16]. Interestingly, people trained in hypnosis had better knowledge about its indications [17] and experience with hypnosis could lead to more openness in anesthesia providers [18]. In fact, knowledge about hypnosis is low in anesthesia providers based on an Australian questionnaire study (N = 218) and an American follow-up (N = 126) [18, 19]. Furthermore, misconceptions regarding hypnoanalgesia are frequent: the practice can be perceived as dangerous, distorting the ability to give consent, being poorly accepted by patients, needing excessive time, and having a limited field of action [18, 19]. Current evidence refutes these beliefs, showing excellent safety when practiced by well-trained practitioners [1, 10, 11]. The patients remain active participants, able to communicate throughout hypnoanalgesia, quite to the contrary as under general anesthesia [20]. A majority of patients accept medical hypnosis [21], and well-trained teams do not need more time for hypnoanalgesia than standard anesthesia [22]. Finally, indications are broad, from long interventional procedures to vascular surgery and neurosurgery [11].

In a perspective of implementing hypnoanalgesia in our European academic anesthesiology division, we had a specific interest in identifying barriers and facilitators amongst our teams. Our aim was to (a) evaluate the interest and knowledge regarding hypnosis in anesthesiology personnel from four large regional or academic hospitals in Switzerland and (b) evaluate any impact on misconceptions of hypnosis training and prior exposure.

## Methods

### Registration and ethical approval

This project, which collected anonymous replies of hospital personnel through a web-based survey, did not fulfil criteria for the Swiss law on human research (Art. 118b, Swiss Constitution, LRH). The need for ethics approval and informed consent was waived by The Cantonal Commission for Ethics in Human Research (Vaud, Switzerland: CER-VD 2023 − 00734). The participants were provided with the rationale for the data collection and the responders gave implicit consent by answering the online questionnaire. No identifying data were collected, and there was no obligation to participate.

### Recruitment and procedure

The anesthesiology divisions of four hospitals in the French speaking part of Switzerland participated between June 2020 and August 2021: Lausanne University Hospital (CHUV), Geneva University Hospital (HUG), Fribourg Regional Hospital (HFR), Valais Hospital (Sion). Of note, Geneva University Hospital had set up an institutional hypnosis program since 2017, training physicians and caregivers of the full institution staff (i.e. 292 trained including 15 physicians and 16 nurses in anesthesiology). Our hypothesis was that this hospital would have more trained providers than those without such a program, although no statistics existed in the other institutions.

An invitation to a survey on the use of hypnosis in the operating room was sent to all physicians and nurses of the anesthesiology divisions through grouped institutional e-mail lists (e.g., “anesthesiology residents”; “nursing anesthesiology team” etc.) by a senior physician from each hospital. This email gave a link to answer a questionnaire on a protected university Redcap server. The distribution started at CHUV (leading house, test-phase June - October 2020) and had to be delayed in the other hospitals due to the covid-19 pandemic. HFR was second February - May 2021; HUG from April 28 until August 1, 2021, and finally the Hospital of Sion from May 7 until June 10, 2021. Three reminders prompting responses were sent to the same mailing lists.

### Exclusion of data

Health providers were invited to respond anonymously and could have participated multiple times, although this seemed unlikely, except by mistake. Two cases of duplicate entry were identified using the SPSS 27 “Duplicate identification function”, followed by a visual check of the original Redcap entries. These entries had identical answers and the same remarks in the open comment box, leading to the exclusion of the duplicates. One responder did not identify as a nurse or physician (checked “other health care professional”), and this entry was excluded.

### Questionnaire

The questionnaire included twenty-one items and took about two minutes to fill (see supplementary table). We translated into French the questions of the “survey of anesthesia provider’s attitudes towards hypnosis” developed in a teaching hospital at Johns Hopkins University, Baltimore, Maryland, USA [19]. The questionnaire included a brief categorical assessment of the responder’s demographics (profession and number of years of practice, exposure to different clinical and show hypnosis). Opinions concerning the role of formal hypnosis and conversational hypnosis (named positive suggestions in the prior study [19]) in the operating room were assessed. It also evaluated common reasons for not using hypnosis: misconceptions (as presented in the introduction: dangerous, ineffective, distortion of consent abilities, poor acceptance by patients, excessive time needed and limited field of action) and practical aspects (the specific training). In the original study, the ratings were dichotomous (agree/don’t agree). To have more detailed results, we modified the questionnaire by using 4-point Likert scales from 1 (absolutely don’t agree) to 4 (absolutely agree); with the possibility to answer “don’t know” on some of the items (see supplementary table).

The usefulness of hypnosis (considering together formal and conversational hypnosis) was rated for different proposed anesthesiology indications (peripartum analgesia, needle phobia, chronic pain, complementary analgesia in minor surgeries). Here too, the original dichotomous reply (useful vs. not) was expanded using a 4-point Likert scale from 1 (not useful) to 4 (very useful).

Furthermore, we added 4 questions to the original questionnaire (see the bold and in italic questions in the supplementary table): we completed the demographic information with the number of years of practice in anesthesiology (categorical 0–3 years, 4 to 8 years, 9 to 14 years; 15 years and more). The participants were asked about an additional, lesser-known indication supported by our clinical practice: i.e. usefulness of hypnoanalgesia instead of general anesthesia for dressing wound closure (4-point Likert scale from 1 (not useful) to 4 (very useful)). The participants reported any experience of medical hypnosis in the OR (Y/N). Finally, the interest in future training (either formal or conversational hypnosis) was assessed (Y/N/maybe/already trained). A space for open comments was also added. Finally, we deleted the question regarding the self-perceived knowledge of hypnosis.

### Statistical analysis

The characterization of the sample was descriptive, reporting the number and the percentage of the respondent by profession, the years of practice and the training in hypnosis. Differences in the proportion of people trained in hypnosis (either formal or conversational) between the four hospitals were assessed with chi-square tests.

A Mann-Whitney U test was performed to evaluate if the level of agreement with the different misconceptions differed by training in hypnosis (trained vs. not-trained) and by hypnosis exposure in the OR (exposed vs. non-exposed). The mean rank for each rating on the 4-point Likert-scale (from totally disagree to totally agree) was calculated and compared across groups; the highest rank corresponded to the highest disagreement with the proposed statement.

An exploratory analysis compared our data with Stone et al [19]: we used chi-square statistics. Since the answers for the Stone and al. survey concerning the barriers to implementation were dichotomous (agree/don’t agree) we transformed our Likert scale results by combining “absolutely agree” & “agree” into “agree”; “absolutely don’t agree” &“don’t agree” into “don’t agree”. This is presented in supplementary materials.

The analysis was conducted with SPSS 27 (IBM, Germany).

## Results

### Study population

Between June 2020 and August 2021, 356 questionnaires were received, and 3 excluded (2 duplicates, 1 non physician/nurse). Therefore, we analyzed 353 answers by anesthesiology health providers of four hospitals (out of N = 754; response rate of 47%), see Table 1 for their full characteristics. The response rate was slightly higher in anesthesiology nurses (N = 206/ 407 invited, 51%) than physicians (N = 147/347 invited, 42%). The responding staff was rather senior (23% with 9–14 years of training, 47% ≥ 15 years).

Amongst the responders, 49 were trained in hypnosis (14%), either in formal hypnosis (N = 10, 3%), conversational hypnosis (N = 19, 5%) or both (N = 20, 6%). Due to their specific training program, the ratio of trained professionals was significantly higher at the Geneva University Hospital (18%) than at the 3 other hospitals (average = 10%), X^2^(1, N = 353) = 4.1, p = 0.04. Overall, the respondents in this sample were largely interested in undergoing conversational hypnosis training (73%) (Table 1). A large proportion of professionals (71%) had been exposed to hypnosis in the operating room.

**Table 1:** Study population

Sample	Total (n = 353)		HUG (n = 169)		3 other Hospitals (n = 184)		X^2^ ; p
Profession (n, %)							*2.5; 0.11*
Physicians	**147**	**42%**	63	37%	84	46%	
Nurses	**206**	**58%**	106	63%	100	54%	
Years of practice							***9.6; 0.02***
0–3	**34**	**10%**	11	7%	23	13%	
4–8	**70**	**20%**	28	17%	42	23%	
9–14	**82**	**23%**	37	22%	45	24%	
> 15	**166**	**47%**	93	55%	73	40%	
Training in hypnosis							***4.1; 0.04***
**Total**	**49**	**14%**	**30**	**18%**	**19**	**10%**	
Formal hypnosis only	**10**	**3%**	6	4%	4	2%	
Conversational hypnosis only	**19**	**5%**	13	8%	6	3%	
Both conversational and formal	**20**	**6%**	11	7%	9	5%	
Interest in hypnosis training							*0.05; 0.81*
**Total**	**257**	**73%**	**124**	**73%**	**133**	**72%**	
Formal hypnosis only	**19**	**5%**	13	8%	5	3%	
Conversational hypnosis only	**113**	**32%**	51	30%	62	34%	
Both conversational and formal	**126**	**36%**	60	36%	66	36%	
Exposure to hypnosis							***4.1; 0.04***
**Any kind of exposure**	**267**	**76%**	**140**	**83%**	**136**	**74%**	
In the operating room	**252**	**71%**	131	78%	121	66%	
For yourself	**91**	**26%**	53	31%	38	21%	
For entertainment	**32**	**9%**	15	9%	17	9%	
Total staff number (Response rate)							***4.5; 0.03***
**Total**	**754**	**47%**	322	52%	429	43%	
Physicians	**347**	**42%**	136	45%	209	40%	
Nurses	**407**	**51%**	186	57%	220	45%	

### Acceptance of hypnosis and specific indications

**Fig. 1:**
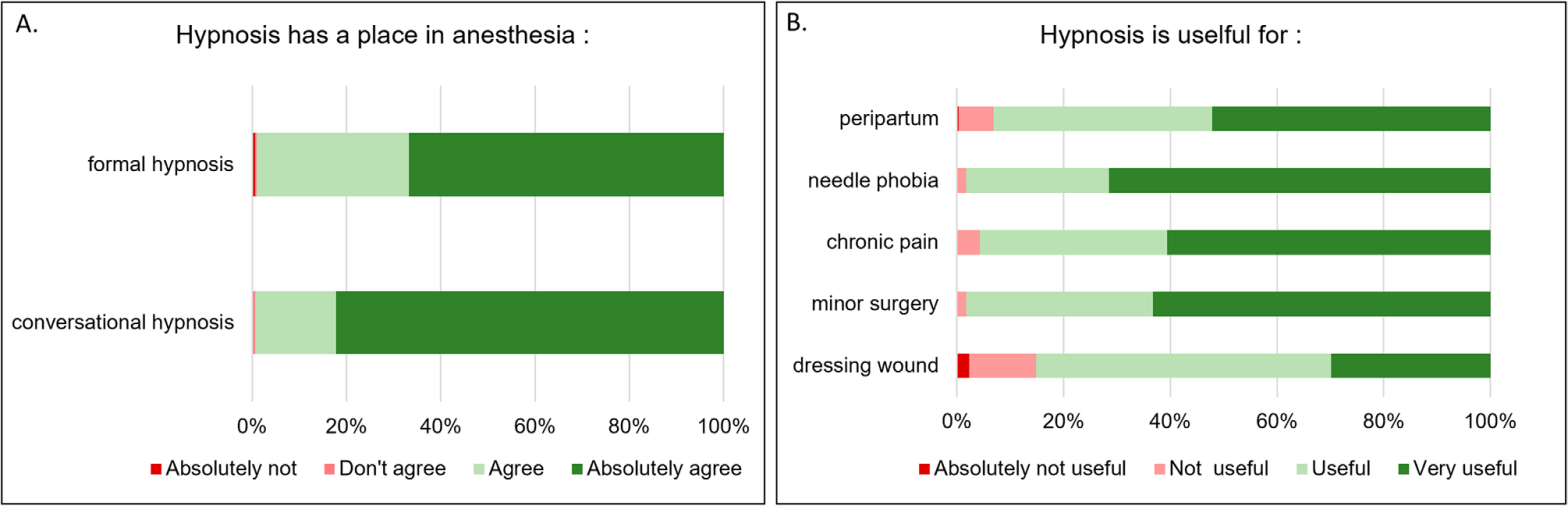
Level of agreement with hypnosis having a role in the practice of anaesthesia (A) and usefulness of hypnosis for different indications (B)

There was a strong agreement for hypnosis having a role in anesthesia (96% agreed for formal and 98% conversational hypnosis) (see Fig. 1A).

There was no difference in acceptance of hypnosis in anesthesia between the HUG (with a training program) and the 3 other hospitals neither for formal hypnosis (U = 15,866, p = 0.11) nor for conversational hypnosis (U = 15,927, p = 0.19). Hence, the analyses were performed on the entire sample, without distinction between the 4 hospitals.

The different indications in the field of anesthesiology had varied levels of agreement (Fig. 1B). The use of hypnosis was judged as non-useful in 14% for dressing wound interventions, 7% in the peripartum, 5% for chronic pain and only 2% for minor surgeries. Usefulness of hypnosis was rated highest for needle phobia (72% judging it as very useful and only 1% thinking it is not useful).

### Perceived barriers and potential effect of hypnosis training and exposure

**Fig. 2:**
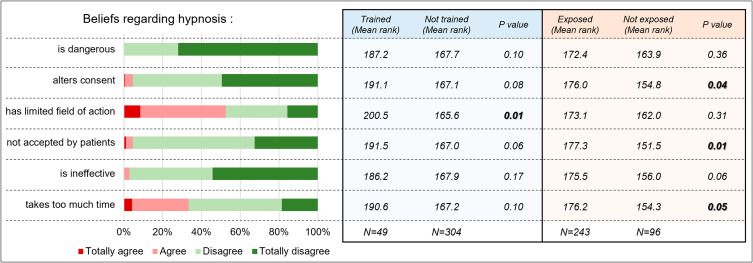
The left side depicts reasons for not using hypnosis, with the percentage of people agreeing with each statement. To the right, two tables present the differences in agreement between trained vs. non-trained respondents (blue, first table) and exposed vs. non exposed respondents (peach, second table). The scores displayed are the mean of the ranks according to the Mann Whitney U test. Higher mean rank reflects higher disagreement with the statement. P values are presented without correction for multiple comparisons

The most frequent reasons for not using hypnosis were: hypnosis needs a special training (92% responders), a perception of a limited field of action (51% responders) and excessive time needed (34% responders) (Fig. 2 left panel). Nevertheless, hypnosis was considered safe (only 1% agreeing that it could be dangerous) and effective (only 3% considering hypnosis as ineffective).

There was only one significant difference in one perceived barrier between trained and non-trained respondents: non-trained people agreed more with the statement that hypnosis has a limited field of action (Fig. 2 right panel, 1st table).

In contrast, there were more differences in misconceptions between people who had been exposed to hypnosis and those not: concerning the alteration of consent, acceptability by patients and the excessive time needed (Fig. 2 right panel, second table).

The only difference that remained after Bonferroni corrections for multiple comparisons shows that non-exposed anesthesia providers were more likely to think patients would not accept hypnosis than the exposed ones.

## Discussion

This survey regarding readiness and barriers to the use of hypnosis in the operating room conducted in 4 Swiss teaching/large regional hospitals’ anesthesiology services collected data from 352 physicians and nurses. The minority of them had a training in hypnosis (14%). The hospital with a specific training program had more trained staff. A majority was interested in gaining at least basic hypnosis training (73%). Most (96%) felt there was a role for formal hypnosis in anesthesiology, with the level of usefulness depending on the specific indication. The major perceived barriers by the anesthesiology teams to the use of hypnosis were of organizational nature (need for specific training) and based on misconceptions (limited field of application, need for excessive time, alteration of consent, unacceptability to patients). These perceptions were not diminished in people trained in hypnosis. However, some were reduced in staff with prior exposure to hypnosis.

### Response, Training and Interest in hypnosis

This anonymous online questionnaire obtained a satisfactory response rate (47%) [23], yet could be subject to response biases. It appears the responders were rather senior (70% in practice since more than nine years), suggesting a bias given the usual pyramidal staff shape in training hospitals. Yet, this provides an interesting insight into the perspectives of health professionals with clinical experience and a potential influential role due to seniority or attending status. One could have expected a bias in response of people trained (and therefore interested) in hypnosis. Our study was initially designed to compare the hospital with a training program to the 3 others. There was a small yet significant difference in training rate. Contrary to our expectations, there were no significant differences in readiness between these hospitals, which could have been due to a ceiling effect (most staff in favor of hypnosis), justifying that no further comparison were tested.

### Misconceptions regarding hypnosis

Hypnosis was seen as an acceptable anesthesiology practice in a vast majority of the surveyed staff. Nevertheless, the breadth of the scope of medical hypnosis (when including formal and conversational) in the peri-operative context is often ignored [24–26].

Hypnosis requesting extra time is also a common misconception. However, interventions under hypnosis instead of general anesthesia have similar or reduced times [1, 22, 27, 28]. The induction of an adequate hypnotic state for hypnoanalgesia takes about 10 min, i.e., the same time as an induction of a general anesthesia [29, 30]. The adjunction of hypnosis doesn’t take any additional time for interventions under local anesthesia such as parathyroid surgery [31]. Also, hypnosis could save time when the eviction of general anesthesia or the reduction of medication leads to a shorter stay in the recovery room [32].

Few survey responders considered hypnosis as dangerous. This seems appropriate. Yet, the potential risks of hypnosis could be minimized due to a lack of knowledge. Adverse reactions have been described such as anxiety or pseudo memories [33]. These are usually attributed to deficiencies in the practice of the hypnotic techniques. Nevertheless, awareness of risks and preparations for a hypnoanalgesia failure can facilitate a smooth conversion to general anesthesia [29, 34]. This remains rare in day surgery, with for example 0,5% conversion rate to general anesthesia in plastic surgery due to surgical complications, anxiety or pain [30].

It is noteworthy that even staff trained in hypnosis can be unaware of hypnoanalgesia’s applications in the operating room and the absence of supplementary time needed by this practice. This could be due to the Swiss medical hypnosis training, which is not specific to anesthesiology and includes few elements relevant to the OR practice. In fact, a reduction in some misconceptions was shown by staff who had been exposed to hypnosis, compared to those not exposed.

### Comparison with prior surveys

Compared to the study on which this survey was based [19], we had a larger, multicentric sample (N = 352 in 4 centers, vs. N = 126 in 1 center). The staff in our sample was more favorable to the use of both formal hypnosis (96% versus 42%) and conversational hypnosis (98% vs. 83%) in anesthesiology (see details in Supplementary Table S3). Less of the Swiss sample had no opinion on the place of formal hypnosis in anesthesiology (3% vs. 47%). Nevertheless, the proportion of staff with some training in hypnosis was not significantly different (mean in our data: 14%; Stone et al.: 13%). Our sample had more prior exposure to hypnosis with 76% reporting previous exposure to hypnosis (of any kind) versus 65% in Stone et al. Interestingly, when looking at barriers to practice, the anesthesia teams in Stone’s study were more often mis-informed, with more of them reporting that hypnosis was ineffective (25% vs. 3%), would not be accepted by patients (21% vs. 5%) than in the present study. Their sample also was more often unaware that hypnosis requires special training (66% vs. 8%).

Hence, the Swiss anesthesiology teams seemed more open and aware of hypnoanalgesia than this US sample [19], as well as another prior Australian sample [18]. This does not appear to be linked with a difference in the training rate, but perhaps to more practical exposure to hypnosis. This would be consistent with prior research in implementation, that theoretical knowledge without application and practical experience have little impact [12].

Furthermore, clinical guidelines and clear protocols regarding hypnoanalgesia are currently lacking. This can be especially problematic in anesthesiology, a protocol-driven specialty. Hypnosis research is also a fast-evolving field, with guidelines and more rigorous study designs aiming to increase impact [35, 36].

The presented results therefore open the path to specific solutions, i.e. favoring targeted interactive educative interventions about hypnosis for all anesthesiology staff to clarify mis-conceptions, allowing first-hand observation of hypnoanalgesia practice [12, 37] and developing specific, hands-on courses for the hypnosis-trained anesthesiology staff. This offer should encounter large interest in staff similar to ours.

## Conclusions

This first European survey of anesthesia providers’ readiness for hypnoanalgesia shows a very positive response in four large Swiss hospitals. The physicians and nurses are motivated to undergo a brief training. The main reasons for not using hypnosis are of practical concern, as well as a few misconceptions. Offering more specific training and especially practical exposure to hypnoanalgesia would favor implementation of this evidence-based complementary practice.

### Electronic supplementary material

Below is the link to the electronic supplementary material.

Supplementary Material 1

## Data Availability

Data and material are available online with a restricted acces (granted upon online request): 10.5281/zenodo.7818137.
