# Supplementary material for: Hypnosis in the operating room: are anesthesiology teams interested and well-informed?
Source: BMC Anesthesiol. 2023 Aug 24;23:287. doi: 10.1186/s12871-023-02229-3 (PMC10464071; doi:10.1186/s12871-023-02229-3)
Supplement: Supplementary file 1 — Supplementary Material 1 [file 12871_2023_2229_MOESM1_ESM.docx]

Supplementary table S1: Listing of the questions asked in the online questionnaire. The translated original questions from Stone et al. are in fine characters, the added questions in bold and italic characters.

| 1. What is your job?  □ Physician  □ Nurse  □ Other health practitionner | | | | | |
| --- | --- | --- | --- | --- | --- |
| ***2. How long have you practiced your profession?***  □ 0-3 years  □ 4-8 years  □ 9-14 years  □ 15 years and more | | | | | |
|  | Absolutely not | Don’t agree | Agree | Absolutely agree | Don’t know |
| 1. Do you agree that formal hypnosis has a role to play in the practice of anesthesia? |  |  |  |  |  |
| 1. Do you agree that conversational hypnosis has a role to play the practice of anesthesia? |  |  |  |  |  |
| 1. Do you agree that hypnosis takes too much time? |  |  |  |  | x |
| 1. Do you agree that hypnosis needs a special training? |  |  |  |  |  |
| 1. Do you agree that hypnosis is ineffective? |  |  |  |  |  |
| 1. Do you agree that hypnosis would not be accepted by patients? |  |  |  |  |  |
| 1. Do you agree that the field of application is limited for hypnosis? |  |  |  |  |  |
| 1. Do you agree that hypnosis distorts the ability to obtain consent? |  |  |  |  |  |
| 1. Do you agree that hypnosis is dangerous? |  |  |  |  |  |
| 1. Do you agree that there are no valid reasons to use hypnosis in your practice? |  |  |  |  |  |
|  | Absolutely not useful | Not useful | Useful | Very useful |  |
| 1. Do you think hypnosis is useful for peripartum analgesia? |  |  |  |  |  |
| 1. Do you think hypnosis is useful for needle phobia? |  |  |  |  |  |
| 1. Do you think hypnosis is useful for chronic pain? |  |  |  |  |  |
| 1. Do you think hypnosis is useful for analgesia in minor surgeries? |  |  |  |  |  |
| 1. ***Do you think hypnosis can usefully replace general anesthesia for dressing wound closure?*** |  |  |  |  |  |
| 1. ***Have you ever observed hypnosis in the operating room?***   □ Yes □ No | | | | | |
| 1. Did you ever experience formal hypnosis for yourself?   □ Yes □ No | | | | | |
| 1. Did you ever attend show hypnosis?   □ Yes □ No | | | | | |
| 1. ***Are you interested in:***   ***- conversational hypnosis training***  □ Yes  □ No  □ Maybe  □ Already trained  ***- formal hypnosis training?***  □ Yes  □ No  □ Maybe  □ Already trained | | | | | |

Supplementary table S2: Detailed study population

| Detailed sample | **Total**  **(n=353)** | | CHUV  (n=102) | | HFR  (n=41) | | HUG  (n=169) | | Sion  (n=41) | |
| --- | --- | --- | --- | --- | --- | --- | --- | --- | --- | --- |
| Profession (n, %) |  |  |  |  |  |  |  |  |  |  |
| Physicians | **147** | **42%** | 57 | 56% | 16 | 39% | 63 | 37% | 11 | 27% |
|  | **206** | **58%** | 45 | 44% | 25 | 61% | 106 | 63% | 30 | 73% |
| Years of practice |  |  |  |  |  |  |  |  |  |  |
| 0-3 | **34** | **10%** | 18 | 18% | 1 | 3% | 11 | 7% | 4 | 10% |
| 4-8 | **70** | **20%** | 24 | 24% | 9 | 23% | 28 | 17% | 9 | 22% |
| 9-14 | **82** | **23%** | 32 | 31% | 9 | 23% | 37 | 22% | 4 | 10% |
| >15 | **166** | **47%** | 28 | 27% | 21 | 53% | 93 | 55% | 24 | 59% |
| Training in hypnosis |  |  |  |  |  |  |  |  |  |  |
| **Total** | **49** | **14%** | **9** | **9%** | **6** | **15%** | **30** | **18%** | **4** | **10%** |
| Formal hypnosis only | **10** | **3%** | 2 | 2% | 2 | 5% | 6 | 4% | 0 | 0% |
| Conversational hypnosis only | **19** | **5%** | 4 | 4% | 1 | 2% | 13 | 8% | 1 | 2% |
| Both conversational and formal | **20** | **6%** | 3 | 3% | 3 | 7% | 11 | 7% | 3 | 7% |
| Interest in hypnosis training |  |  |  |  |  |  |  |  |  |  |
| **Total** | **257** | **73%** | **78** | **76%** | **24** | **59%** | **124** | **73%** | **31** | **76%** |
| Formal hypnosis only | **19** | **5%** | 3 | 3% | 0 | 0% | 13 | 8% | 2 | 5% |
| Conversational hypnosis only | **113** | **32%** | 34 | 33% | 13 | 32% | 51 | 30% | 15 | 37% |
| Both conversational and formal | **126** | **36%** | 41 | 40% | 11 | 27% | 60 | 36% | 14 | 34% |
| Exposure to hypnosis |  |  |  |  |  |  |  |  |  |  |
| **Any kind of exposure** | **267** | **76%** | **70** | **69%** | **33** | **80%** | **140** | **83%** | **33** | **80%** |
| In the operating room | **252** | **71%** | 63 | 62% | 29 | 71% | 131 | 78% | 29 | 71% |
| For yourself | **91** | **26%** | 23 | 22% | 7 | 17% | 53 | 31% | 8 | 20% |
| For entertainment | **32** | **9%** | 7 | 7% | 5 | 12% | 15 | 9% | 5 | 12% |
| Total staff number (Response rate) |  |  |  |  |  |  |  |  |  |  |
| Total | **754** | **47%** | 229 | 44% | 115 | 36% | 322 | 52% | 85 | 48% |
| Physicians | **347** | **42%** | 131 | 43% | 41 | 39% | 136 | 45% | 37 | 30% |
| Nurses | **407** | **51%** | 98 | 46% | 74 | 34% | 186 | 57% | 48 | 62% |
| CHUV: Lausanne University Hospital; HFR: Regional hospital of Fribourg; HUG: Geneva University Hospital; Sion: Valais hospital | | | | | | | | | | |

*Supplementary Table S3. Comparison regarding beliefs about hypnosis in the operating room between our pooled sample of 4 Swiss hospitals and a prior American study (Stone et al.) [18].*

|  | | Pooled sample of current study  (N=353) | | Stone et al.  (N=127) | | | *P value* | | *X^2^* |  |
| --- | --- | --- | --- | --- | --- | --- | --- | --- | --- | --- |
| Formal hypnosis has a role to play in the practice of anesthesia? | Absolutely not | 2 | 1% | | 3 | 2% | |  |  | |
|  | Don’t agree | 1 | 0% | | 12 | 10% | |  |  | |
|  | Agree | 111 | 31% | | 42 | 33% | |  |  | |
|  | Absolutely agree | 229 | 65% | | 11 | 9% | | *<0.01* | *107.8* | |
|  | Don’t know | 10 | 3% | | 59 | 47% | | *<0.01* | *151.4* | |
| Conversational hypnosis has a role to play in the practice of anesthesia? | Absolutely not | 0 | 0% | | 6 | 5% | |  |  | |
|  | Don’t agree | 2 | 1% | | 2 | 2% | |  |  | |
|  | Agree | 60 | 17% | | 57 | 45% | | *<0.01* | *38.2* | |
|  | Absolutely agree | 286 | 81% | | 48 | 38% | | *<0.01* | *80.7* | |
|  | Don’t know | 5 | 1% | | 14 | 11% | | *<0.01* | *25.2* | |
| Hypnosis has a limited field of action | Agree | 185 | 53% | | 20 | 16% | |  |  | |
|  | Don’t agree | 167 | 47% | | 106 | 84% | | *<0.01* | *46.7* | |
| Hypnosis takes too much time | Agree | 177 | 33% | | 52 | 41% | |  |  | |
|  | Don’t agree | 233 | 67% | | 74 | 59% | | *ns* | *2.4* | |
| Hypnosis would not be accepted by patients | Agree | 16 | 5% | | 27 | 21% | |  |  | |
|  | Don’t agree | 331 | 95% | | 99 | 79% | | *<0.01* | *26.9* | |
| Hypnosis distorts the ability to obtain informed consent | Agree | 16 | 5% | | 13 | 10% | |  |  | |
|  | Don’t agree | 332 | 95% | | 113 | 90% | | *ns* | *5.3* | |
| Hypnosis is ineffective | Agree | 10 | 3% | | 31 | 25% | |  |  | |
|  | Don’t agree | 339 | 97% | | 95 | 75% | | *<0.01* | *50.4* | |
| Hypnosis is dangerous | Agree | 1 | 0% | | 6 | 5% | |  |  | |
|  | Don’t agree | 348 | 100% | | 120 | 95% | | *ns* | *3.2* | |
| Hypnosis needs a special training | Agree | 322 | 92% | | 43 | 34% | |  |  | |
|  | Don’t agree | 27 | 8% | | 83 | 66% | | *<0.01* | *174.2* | |
| Staff trained in hypnosis |  | 49/353 | 14% | | 18/137 | 13% | | *ns* | *0.1* | |
| Staff exposed to hypnosis (any kind) |  | 276/353 | 76% | | 89/137 | 65% | | *<0.01* | *9.9* | |
